# What constitutes a quality community aged care service—client perspectives: An international scoping study

**DOI:** 10.1111/hsc.13998

**Published:** 2022-09-09

**Authors:** Sandra Smith, Melinda Martin‐Khan, Catherine Travers

**Affiliations:** ^1^ Centre for Health Services Research The University of Queensland Brisbane Queensland Australia; ^2^ School of Health and Rehabilitation Sciences The University of Queensland Brisbane Queensland Australia

**Keywords:** community services for the elderly, community‐based research, home care, older people's services, patient preferences, patient satisfaction

## Abstract

Overwhelmingly, older Australians (people aged 65 years and older or 50 years and older for Aboriginal and Torres Strait Islander people) prefer to remain living in their own home rather than moving into residential care. To enable older Australians who require assistance to remain living at home, the Federal Government provides a wide range of community care services, the provision of which has increased substantially over the last 15 years. The importance of client preferences, prefaced by the introduction of consumer directed care across community aged care services, has gathered momentum in Australia following legislation in 2015. Older peoples' preferences differ in comparison to younger people with disability and those with mental health concerns. Older people focus more on the provision of services rather than the notion of independent living itself. This scoping review aimed to explore what aspects people aged 65 years and older consider to be important qualities of aged care services delivered in the community. A computerised search in MEDLINE, CINAHL, PubMed and PsychINFO and hand searches of the Cochrane database and Google Scholar were completed in May 2022. Sixty‐two articles met the selection criteria and were included in the review. Data were extracted using a fit‐for‐purpose protocol and analysed using the Miles and Huberman Model for thematic analysis. Results identified five themes representing quality domains that describe features that are important to clients: staff knowledge, respect for clients, a person‐centred approach, a collaborative partnership with clients and clear communication. When providers of community aged care services are planning to assess the quality of their services, these findings could be used to guide their evaluation. This will ensure that future services delivered accommodate the needs and preferences of clients who receive them.

**What is known about this topic**

- Quality measures are a pre‐requisite to improving the quality of care by enabling ongoing monitoring and comparing performance against a set of standards and/or measures.
- Many aged care service providers use satisfaction surveys to measure their clients' satisfaction.
- Very few aged care service providers develop satisfaction surveys in partnership with their clients, to ensure the views and opinions of features important to clients are included.

**What this paper adds**

- The views and preferences of older community aged care service users regarding the provision of a quality service were identified through an international scoping literature review.
- A suite of quality domains that could be used by aged care providers to measure quality from the client's perspective when delivering services in the community.

## 1 INTRODUCTION

Individual experience is considered one of the keys to quality in healthcare, along with safety and clinical outcomes. Providing feedback on the experience of clients and supporting the organisational team to address any identified problems, leading to improved quality, is the best way to support a service to meet its objectives (Santana et al., 2019). However, despite the legislated collection of client feedback, its use by healthcare leadership is still limited (Santana et al., 2019). According to Donabedian (1988), the definition and scope of the word ‘quality’ is significant, such that, before we even begin to measure quality, we need to understand what elements constitute quality. These elements are dependent upon the nature and the extent of the responsibilities of the service being evaluated. Furthermore, Donabedian (1988) identifies the importance of the contribution of interpersonal relationships, being the vehicle enhancing the delivery of technical care, upon which successful outcomes ultimately depend. Aged care services provided in the community involve several interpersonal relationships, including those between clients, support workers and service providers, yet technical aspects of care provided are often the focus of quality reporting across the sector, and preference is given to client satisfaction surveys to reflect client views regarding the services provided (Gill et al., 2011). Consumer directed care has been the focus in both Australia and internationally by way of promoting choice and autonomy in the way older people choose to live their lives in the community, including their choice of the care and services they receive (Commonwealth of Australia, 2009). Furthermore, the National Health and Hospital Reform Commission (NHHRC), 2010, in its report, ‘National Health and Hospital reform commission final report, and patient‐centred suggestions for reform’, explicitly identified the need for future health reform to improve the experiences of the population groups they serve, by including their voices to direct priorities for future reform (Jowsey et al., 2011). There are three main government objectives for Australian aged care services including accessibility, appropriate to the needs of clients and high quality. A framework of performance indicators is based on these three objectives with the intention of providing information about the equity, efficiency and effectiveness to identify the outputs and outcomes of aged care services (Australian Government Productivity Commission, 2021). Current performance indicator measures defined under the quality objective that relates to client feedback include satisfaction (satisfaction with the range and quality of both formal and informal services provided) and complaints (received by the Aged Care Quality and Safety Commission). Compliance with the Aged Care Quality Standards introduced on 1 July 2019 is an additional performance indicator defined under quality. The government objective intended to measure the appropriateness of services addressing a client's need has no data available to date. The intent of this objective is to measure the extent to which the client and/or their representative had input into planning their care, whether the supports identified in the care planning process were provided and to what extent the interests, customs, beliefs and cultural and ethnic backgrounds of clients were valued and respected (Australian Government Productivity Commission, 2021). In Australia, organisations deliver both federally funded and privately funded services to older Australians living in the community and in residential aged care settings. Federally funded services provided to support clients with care needs to remain living in their own homes include low‐level services (Commonwealth Home Support Program) and higher‐level care services (home care package). The use of both services (CHSP and HCP) has grown substantially over the past 10 years with a corresponding increase in the number of organisations providing services, while demand for residential aged care has declined (Australian Government Productivity Commission, 2021).

A scoping literature review was undertaken to explore current international research on the elements clients consider to be important for community aged care services. Aged care services delivered in older people's homes and the community are described and delivered in very different ways, depending on the legislation that governs their delivery in each country. These differences include the assessment criteria used to determine a clients' eligibility to access services, the financial contribution required of clients when receiving services, and the regulatory requirements of individual service providers who deliver these services. Including the voices of older clients living in the community who are receiving aged care services is an integral part of service delivery (Zhou et al., 2021). Currently, there is limited published literature on client perspectives regarding aspects of service quality such as eligibility, financial contributions and the quality of services. This includes information regarding the quality of the gatekeeper service—in Australia, it is the Aged Care Assessment Program that assesses eligibility for services. The aim of this review was to identify client‐derived quality measures relevant to government‐funded health policy initiatives (dated no later than 2000) to operate community aged care services. While it is recognised that there are country‐specific differences, this scoping review focussed on the views and preferences of clients in relation to the qualities they consider to be important in an aged care service, that was delivered in a community setting.

## 2 METHOD

A systematic search of electronic databases: MEDLINE, CINAHL, PubMed and PsychINFO were concluded in May 2022, including a targeted search of the Cochrane database and Google Scholar. In accordance with the Australian aged care system, age requirements (> 65 years or >50 years for Aboriginal and Torres Strait Islander people), two separate systematic searches were undertaken, specifying the difference in age categories and population cohorts only. Filters were applied to the electronic searches including limiting the search to academic journals published between July 2000 and April 2022, age (65 years and over and 50 years and over for Aboriginal and Torres Strait Islander people) and human. The year 2000 was selected as the commencement date as publications prior to this date would not reflect current Australian policies and practices. Duplicates were removed, and titles/abstracts were reviewed by one author (SS). Full texts were independently reviewed by three authors (SS; and either CT or MMK). Disagreement was resolved by consensus in the discussion. Full‐text articles were included if there was a reference to (a) either a client, patient or lived experience expert; (b) community aged care services or home care services and (c) quality measure or satisfaction or preference or participation (Table 1). Data analysis using the 4‐step Miles and Huberman's model (1994) for thematic analysis was carried out on the included final articles. Thematic analysis was chosen as the method for interpreting the data due to its flexibility including, the research question of the study, the constitution and size of the sample and the data collection method used (Clarke & Braun, 2017).

**TABLE 1:** Inclusion and exclusion criteria for selection of relevant publications

Inclusion	Exclusion
Client (>45 years Aboriginal and Torres Strait Islander cohort) involvement in project or opinion reported in results	Professional or clinician perspective (no client >45 years Aboriginal and Torres Strait Islander perspective) Opinion reported (second‐hand opinion of assumed public preference, rather than a direct report of public/client data)
Client (>65 years) involvement in project or client opinion reported in results	Professional or Clinician perspective (no client [>65 years] perspective) Opinion reported (second‐hand opinion of assumed public preference, rather than a direct report of public/client data)
Community aged care service OR home care service	Residential aged care, hospital, residential respite care, outpatients, day clinic, GP practice
Measure of quality: measure of client satisfaction OR participation OR viewpoint OR preference	Focus on demographic data or service data (e.g., length of stay, cost, efficiency of service or staffing)
Outcome measurement	Abstract not available in English

### 2.1 Step 1—Data reduction

The first stage of data reduction was conducted according to step 1 of Miles and Huberman's model (1994) for thematic analysis (Ibrahim‐Alhojailan, 2012). Data were extracted from the final articles using a protocol designed by the first author specifically for this purpose. Data elements included: publication date, authors, country, sample/population group, study design, methods used, aims, outcomes, service type and key measures informing results. In line with the aim of the review, detailed extraction occurred, where there was information regarding client (patient) involvement in the planning and development of study protocols and/or development of the study, including pre‐ and post‐data collection. Some studies were inclusive of clients in age groups that were outside of the exclusion criteria of this scoping review and included quantitative data in their results. For the purposes of this study, qualitative data of client's responses in the age groups nominated for inclusion in this scoping review were extracted only. All analyses were performed by the first author (SS) and reviewed by the other authors (CT and MMK), and any disagreements were resolved by discussion.

### 2.2 Step 2: Identifying themes (validating and establishing reliability)

Data were initially categorised into three broad groups: the aim(s) of the study, methods adopted and key measures used to obtain study outcomes (Ibrahim‐Alhojailan, 2012). Data were further simplified to enable conclusions to be drawn. Themes were developed following the identification of recurring patterns in the data. This process involved the reduction of the aims (perceived impact on clients, perception and experiences of clients and client satisfaction), methods adopted (questions asked of clients) and outcomes (frequency of client quotes relating to impact, experience and satisfaction).

### 2.3 Step 3: Data display

Utilising the data extracted from each article in step 1 and summarised as ‘key measures used to obtain study outcomes’ (Ibrahim‐Alhojailan, 2012) (Tables 3, 4, 5, 6, 7); a third step was undertaken in which similar themes were grouped with a focus on topic rather than method (forming key attributes), which informed definitions of each key quality measure (referred to as quality measures used in included papers to obtain outcomes of the study), from the client's perspective (Table 2).

**TABLE 2:** Definitions of key measure of quality and links to relevant included studies from scoping literature review search

	Definition (as described by clients)	Relevant Publications (Table, First Author^#^)
Client satisfaction	Being able to make decisions about the care received, having sufficient time to talk to staff, being treated with dignity and respect, receiving care and support from competent and knowledgeable staff and being treated as an equal person in the care relationship and having positive interpersonal relationships with staff	Table 3: Mason^1^; Bikker^2^; Aletras^3^, Anderson^4^, Chesterman^5^, Royal Commission into Quality of Aged Care^6^, Byrne^7^, Kajonius^8^, Bennett^9^, Skaperdas^10^, Kwak^11^, Eloranta^12^
Client experience	Maintaining personal autonomy, equality in the care relationship, consistent and appropriate care that meets individual's needs, having choice over the care provided, being provided with information that is easily understood to facilitate informed decisions about care and being empowered to contribute to their own care	Table 4: Day^13^, Doyle^15^, Day^16^, Petriwsky^18^, Mahoney^19^, Gill^20^, Gill^21^, Fraser^23^, Teale^24^, Janlov^25^, Pejner^26^, Rabiee^27^, Spoorenberg^28^, Wilde^29^, Simons^30^, Tiilikainen^31^, Russell^32^, Compton^34^, Wennman^35^, Askew^36^
Access and choice	Receiving appropriate information to inform choice (including consumer experience measures), having choice and control over daily life (including the ability to maintain social connections with their community, spirituality, cultural, sexual and religious identity), support and information to understand how care relates to them, being informed of the different types of care available, to have the opportunity to be able to engage in their own care (inclusive of those with language and communication difficulties)	Table 5: Council on the ageing Australia^32^, Sefcik^32^, Suurmond^34^
Preference and expectation of care	Having opportunities for connectedness and engagement in social and recreational activities, flexible care plans that accommodate individual preferences, choice of support workers who provide care, choice over what care types are delivered with funds allocated, being treated with dignity and respect, staff who are well trained and knowledgeable, care should be personalised and adapted to client's s individual preferences and needs	Table 6: Chester^35^, Kaambwa^36^, Abbott^38^, McCaffney^39^, Low^41^, Laragy & Vasiliadis^42^, Sanerma^43^, Wang^44^, Holloway^45^, Lukaszyk^48^
Service quality	Suitable times for the delivery of services, services delivered by professional staff who treat them with dignity and respect, being treated as an equal partner in the care relationship and being involved in the decision‐making process, highly trained staff, care that meets client's needs and is provided by staff who see them as a person beyond their care needs, care that is reliable and consistent	Table 7: Jones^42^, Firbank^43^, Gregory^44^, Samuelsson^45^, Cohen‐Mansfield^46^, Gorenewoud^47^.

### 2.4 Step 4: Drawing conclusions from the data

Conclusions were drawn by taking the key attributes of the client's perspectives that informed the definitions for each key quality measure in Step 3 (Table 2) and grouping these key attributes together by their likeness in meaning (Ibrahim‐Alhojailan, 2012).

## 3 RESULTS

### 3.1 Data reduction

#### 3.1.1 Selection and extraction of data

**Figure 1:**
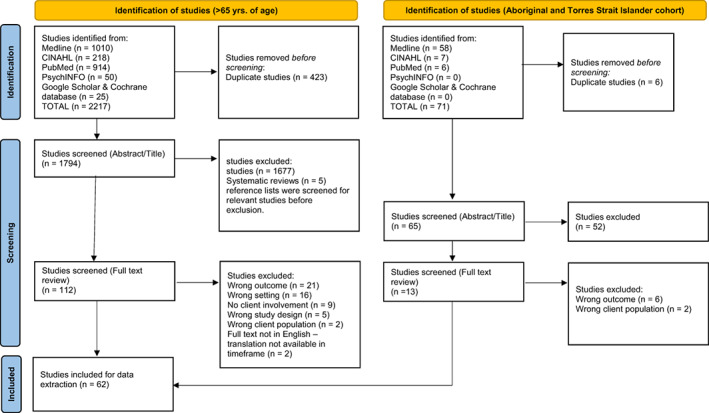
Flow diagram for study selection

The search initially identified 2288 articles which decreased to 125 at abstract levels, including five literature reviews. The five literature reviews were excluded although their reference lists were scanned for relevant articles. Relevant full texts were reviewed and if they met the inclusion criteria were added to the full‐text articles for inclusion. The final 62 articles included seven additional articles that were identified following scanning of the reference list (Figure 1).

#### 3.1.2 General paper details

The largest number of publications were from Australia (*n* = 22), followed by the United Kingdom (*n* = 8), United States (*n* = 7), Sweden (*n* = 6) and Canada (*n* = 5). The remaining articles were published in the Netherlands (*n* = 3), Finland (*n* = 3) and Greece (*n* = 2) and one was from each Korea, Switzerland, Israel, Norway, Hong Kong and Scotland.

All articles discussed the delivery of a broad range of services such as personal care, domestic assistance, transport and community access, social support, day‐to‐day assistance with shopping and meal preparation, nursing, allied health and specific community programs such as fall prevention and palliative care.

The study population for most articles was similar, most often being older people living in the community, however, three articles focussed on support services for people living with dementia (Chester et al., 2018; Fæø et al., 2020; Low et al., 2013), five on Aboriginal and Torres Strait Islander cohorts (Angell et al., 2018; Askew et al., 2016; Holloway et al., 2015; Lukaszyk et al., 2017 & Swain & Barclay, 2015), one on older people with mental illness (Mason et al., 2004), one on older people living in low‐income areas (Kwak et al., 2017) and two on services for the Veteran community (Jones et al., 2007; Mahoney et al., 2019). The inclusion criteria did not specify whether studies were to be qualitative for inclusion, however, 81% (*n* = 50) of the included studies were qualitative. The remaining studies were mixed methods (qualitative and quantitative) (*n* = 3), Discrete Choice Experiment and cross‐sectional (*n* = 1), randomised control trial (*n* = 1), non‐randomised control trial (*n* = 1) and discrete choice experiment (*n* = 3).

#### 3.1.3 Client involvement in the design of methodology

Six of the 62 articles included in this scoping review engaged clients in certain phases of the study, including involving clients in the design of focus group questions, inviting clients to be members of a reference group, involving clients in consultations that were led by clients, involving clients in the development of questionnaires, patient‐reported experience measures and a Discrete Choice Experiment survey (Aletras et al., 2010; Angell et al., 2018; Chester et al., 2018; Groenewoud et al., 2008; Mason et al., 2004; Teale & Young, 2015). Qualitative data collected from clients were described in the articles as occurring primarily by focus groups, interviews or surveys (Tables 3, 4, 5, 6, 7). One study used a yarning circle which is a process used in Aboriginal and Torres Strait Islander culture (Lukaszyk et al., 2017).

**TABLE 3:** Key methodological details and results of included studies measuring client's satisfaction with care provided; quality of life and psychological well‐being (*n* = 15)

Study: Author, Country	Population	Aims	Methods	Key measure and service type	Key findings
Mason^1^ et al. (2004), United States	Mental health consumers with severe and persistent mental health concerns receiving services in three urban mental health centres Consumers: *n* = 42, providers: *n* = 19 (age range > 15–70 years)	To identify factors that contribute to consumer satisfaction. Key Question: What is the gap between what consumers want and what providers can provide?	Qualitative. Focus groups (consumers: *n* = 7 focus groups, providers: *n* = 3 focus groups [from three different providers]) Semi‐structured interview questions were developed by four providers, one consumer and one program evaluator	Service type: Mental health centre Measure: satisfaction	Results were reported for all age groups combined: consumer–provider relationship (reliable access, punctuality, ability to communicate, appointments at a convenient time), competence and knowledge of provider (providers helps consumers understand what the problem is, knows what helps/does not help, believes in recovery, knows about tangential issues) and cultural and religious competence of providers
Bikker^2^ and Thompson (2006), Scotland	People aged >16 years, residing in Scotland in 2000 (*n* = 3052) receiving four different community services domiciliary care (*n* = 296), GP, hospital (outpatient)	To obtain an overall measure of patient satisfaction across different service models of care and to inform policymakers and managers	Qualitative, general population surveys; secondary data analysis of national telephone survey Questionnaires related to the use of services in the last 12 months	Service type(s): domiciliary, GP, outpatient Measure: satisfaction	Results for older people (>65 years) who were previous users of domiciliary care (*n* = 74; 25%): acceptable waiting time for an appointment and convenience of visiting times (domiciliary care) and privacy respected and being treated with dignity (domiciliary care)
Aletras^3^ et al. (2010), Greece	Consumers aged 65–90 yrs enrolled in five home care programs, *n* = 201	To develop and psychometrically test a Greek language instrument for measuring, satisfaction with home care	Qualitative: 31‐item Questionnaire developed following a literature search and onsite observation cognitive interviews (*n* = 15 consumers, *n* = 15 employees), questionnaires delivered via face‐to‐face interviews	Service type: home care Measure: satisfaction	Socio‐economic change staff skills and attitudes: staff demonstrate respect and understand clients' needs appropriateness of provided services: staff forces client to do things he/she dislikes, client trusts staff for providing services and planning of services: inadequate frequency of visits, suitability of scheduled days and scheduled time of visits
Anderson^4^ et al. (2006), America	People with disability receive Medicaid‐financed home and community‐based services in six states Data extracted for home care clients >65 years only (*n* = 612 consumers)	To assess the impact of workforce issues (recruitment, retention and training) on two measures of consumer satisfaction (treatment and respect) with personal assistance services	Qualitative. Computer‐assisted telephone interviews, satisfaction with paid personal assistance scale (8‐question scale)	Service type: home care (personal‐assistant services) Measure: satisfaction vs workforce issues	Workforce issues: (>65 years data only) workforce issues take precedence over health and functional status domains, problems with worker recruitment (e.g., replacing a worker and dealing with unscheduled absences) were more important indicators of consumer dissatisfaction than problems with retention (e.g., having many different workers over time), consumer perception of workers' skills and competency (having well‐trained workers was valued as much as having no recruitment problems) and worker mistreatment of consumers indicator of dissatisfaction (staff treating consumers with respect)
Chesterman^5^ et al. (2001), United Kingdom	Consumers of 10 local authorities in England and Wales receiving community care. Clients >65 years. (*n* = 1268)	To assess satisfaction with outcomes, focussing on equity and efficiency in post‐reform community care at two time points (at the time of offer of service and 6 months later) and the relationship with consumer characteristics	Qualitative. Two time points T1 = immediately after assessment; T2 = 6 months post. Interviews completed T1 (case managers and service users, *n* = 495). Interviews completed T2 (6 months post, users only, *n* = 224)	Service type: home care service Measure: satisfaction	Five key relationships were identified between satisfaction outcomes and the following factors: user characteristics (e.g., user needs such as physical and mental health), carer characteristics, resource levels (e.g., social workers as case managers, meals, access to daycare [centre‐based respite] and respite; location of services; life satisfaction) Overall findings: Expressions of satisfaction are related to ‘objective’ aspects of services but are also a function of user and carer circumstances and characteristics that have little or nothing to do with the receipt of services
Royal Commission into the quality of aged care^6^ (2021), Australia	Australian consumers accessing Commonwealth government aged care services. Home care package clients (*n* = 865) Commonwealth Home support program clients (*n* = 155) Residential respite clients (*n* = 203) NB: over half were client representatives on behalf of clients	To explore the quality of care, general life satisfaction, quality of life (QoL), concerns and complaints of consumers of aged care in Australia. Key question: What do consumers feel about their lives and the care they receive	Qualitative. Computer‐assisted telephone interviews	Service type: home care Measure: life satisfaction, quality of life, quality of care, complaints	Home care Package (HCP) results: levels 1–4, where level 4 is the highest care package Quality of care Receiving daily‐living support was significantly more positive with increasing levels of HCP 2 General life satisfaction Decreased from level 1 to level 4; inner regional areas had higher general life satisfaction compared to metropolitan areas; regional areas fell in between 3 General QoL Scores were recorded as lower when completed by proxy (carer); higher quality of life rating for HCP levels 1 and 2 in comparison to levels 3 and 4; major cities had lower quality of life compared to inner and outer regional areas 4 Social care‐related QoL decreased with increasing levels of HCP; metropolitan cities had lower scores in comparison to inner and outer regional areas 5 Concerns raised by consumers number of concerns increased with HCP level; increase pattern of concerns when interviewed proxy (carer); Concerns included: finance and administration, staff, choice, service type, being treated with dignity and respect Concern with high importance finance (value for money, transparency of fees and charges, unable to negotiate costs)
Byrne^7^ et al. (2011), Canada	Clients >65 years. living at home or in assisted living, receiving private and public home care services Older adults *n* = 82; family members *n* = 52	To understand the reasons for contingent responses about home care from older consumers	Qualitative; face‐to‐face interviews; semi‐structured interview questions	Service type: home care Measure: satisfaction	Individual staff: adept vs inept staff (important to have consistently ‘good’ staff); scheduling of home support: predictable vs precarious scheduling (timing of support visits and continuity of care provided) and types and quantity of services received: responsive versus restrictive care plans (satisfied with services received, not satisfied with restrictions placed on care plans that did not meet care needs)
Kajonius^8^ and Kazeemi (2016), Sweden	Nationwide survey on Eldercare. 324 Swedish municipalities and districts Home care clients >65 years. (*n* = 57, 687)	To explore the effects of structure and process on care satisfaction by older people—measured using Donabedian's (1988) model of quality of care	Qualitative, Survey questionnaire	Service type: Home care Measure: Satisfaction	Impact on client satisfaction: Process‐related factors (respect, influence and information) were more strongly associated with older persons' satisfaction in home care over structural factors (budget, training and staff)
Bennett^9^ and Riedel (2013) Switzerland	Clients >70 years. living at home, receiving formal and informal care (*n* = 712)	To what extent is general life satisfaction of people ≥70 years affected by the existence of a particular care and care arrangement, age and strength of the social network	Qualitative, Standardised survey, face‐to‐face interviews; logistic regression analysis	Service type: Home care Measure: life satisfaction	Impact of care on client life satisfaction required and used home care does not predict life satisfaction, high life satisfaction is possible, even with severe limitations (If people are satisfied with the care they receive, do not have pain and have regular social contacts), those with the strongest social network are most satisfied and a mix of formal and informal care is very important to enable patients to stay in their own homes without relatives burning out
Skaperdas^10^ et al. (2010), Greece	Older people receiving help from the help at home program (*n* = 300)	To investigate older people's satisfaction with the ‘Help at Home’ programme in a prefecture of central Greece	Qualitative, face‐to‐face interviews using a questionnaire based on literature findings; Likert scale	Care type: Home care Measure: satisfaction	Older people satisfaction: High satisfaction having positive interpersonal relations with staff, sufficient system (staff come at pre‐arranged time) and more visits and more hours provided Low satisfaction limited range of services offered (limited programme structure and choice of services)
Kwak^11^ et al. (2017), Korea	Older people aged >65 years. receiving welfare benefits and care in nursing homes and the community. Home care data (*n* = 123)	To assess satisfaction with care in a nursing home and at home among low‐income elders in South Korea.	Qualitative, cross‐sectional study. Face‐to‐face interviews, Questionnaires based on Consumer Quality Index	Care type: Home care Measure: Satisfaction	Satisfaction (homecare group): Highest satisfaction rating: quality of caregivers (including friendliness, safeguard and reliability). Lowest satisfaction rating: meals
Eloranta^12^ et al. (2010), Finland	Older people receiving home care services aged between 67 and 96 years. (survey distributed to *n* = 200) and professionals providing services (survey distributed to *n* = 570)	To explore and compare older home care clients' (65+) and their professional's perceptions of the clients' psychological well‐being and care and to identify possible differences	Qualitative, descriptive survey design, postal questionnaire Surveys returned from 120 home care clients and 370 professionals	Care type: Home care Measure: satisfaction (care and psychological well‐being)	Satisfaction clients were generally satisfied with physical care (medication, self‐care, skin care and house care), clients were less satisfied with psychological and social care (e.g., staff not recognising loneliness and isolation) and clients were dissatisfied with the support of professionals concerning depression/loneliness. Clients rated the following poorly: staff motivate clients to engage in activities outside the home and relevant community groups, staff motivate clients to communicate with other people, staff motivate clients to engage in hobbies/activities and staff motivate relatives to participate in care
Cleland^13^ et al. (2021) Australia	People aged ≥65 receiving a home care package from five providers in metropolitan and rural Australia (*n* = 41, age range 68–95 years.) Eligibility: able to communicate in English, no mild cognitive impairment/mild dementia and ability to provide informed consent	To understand the important quality of life characteristics of older people in receipt of a home care package in Australia	Qualitative study, purposive sampling. Face‐to‐face, semi‐structured interviews using open‐ended questions and 12 cards to probe participants about dimensions of quality of life. Twelve cards detailed descriptors based on EQ‐5D, AQoL and the ASCOT	Care Type: Home care Measure: Impact of home care on Quality of Life	Five dimensions identified that impact Quality of Life for older people: (1) independence, (2) social connections, (3) emotional well‐being, (4) mobility and (5) activities
Lämås^14^ et al. (2021), Sweden	Older people (age range 65–100 years.) (receiving home care services in Sweden) completed survey (*n* = 81): intervention group (*n* = 40) and control group (*n* = 41). staff completed survey (*n* = 48): intervention group (*n* = 19) and control group (*n* = 29)	To examine the effects of a person‐centred and health‐promoting intervention compared to usual care on older people receiving home care services, health‐related QoL thriving and self‐determination	Non‐randomised control trial with a before/after design. Intervention group (person‐centred care intervention implemented), control group (usual care)	Care Type: Home care Measure: Older persons QoL with person‐centred and health promotion home care services	Client results from baseline to follow up: Thriving: older people reported maintaining high levels of thriving in the intervention group versus a decline over time in the control group Negative emotions: older people in the intervention group reported an increase in negative emotions in comparison to the control group
Jones^15^ et al. (2007), United States	Veterans, receiving primary care visits in the community, with experiences of homelessness (age range 18–>66 years.) total surveys (*n* = 5766), total surveys analysed excluding missing data (*n* = 4777)	To evaluate veterans with homeless experiences use of and satisfaction with community care	Qualitative study. Cross‐sectional analysis of responses to a national survey (Primary Care Quality Homeless Services Tailoring Study) Survey Survey measures: self‐reported use of home care, satisfaction with home care and experiences with home care (using Primary Care Quality Homeless survey)	Care Type: Home care Measure: Satisfaction	Three hundred and twenty‐one (19.7%) of samples were aged >65 years and reported using community care Older veterans, females, or who had greater social support as more likely to be satisfied with the community care received Veterans with travel difficulties were less likely to be satisfied with community care and timeliness of care Veterans receiving community care without a veteran coordinator service reported lower satisfaction with coordination of and access to community care

Abbreviations: AQoL, Assessment of Quality of Life; ASCOT, Adult Social Care Outcomes Toolkit; EQ‐5D, European Quality of Life—5 Dimensions; GP, General Practitioner.

**TABLE 4:** Key methodological details and results of included studies measuring clients experience in relation to empowerment, impact of care, service changes, delivery of services and care and participation (*n* = 24)

Study: Author, country	Population	Aims	Methods	Key measure and service type	Key findings
Day^13^ et al. (2017), Australia	Clients aged 81–90 years., living in the community, receiving a home care package *n* = 123 clients invited to participate, *n* = 5 responded	Explore perceptions and experiences of older people using home care packages prior to the introduction of consumer directed care	Qualitative Face‐to‐face in‐depth interviews Semi‐structured interview guide, using reflective journaling, palm cards with emotional touch phrases and field notes	Service type: Home care package Measure: perceptions and experience	Seeking quality and reciprocity in carer relationship: patchworking of services (making it work), the waiting game (waiting for the package) and clients want technology with utility (i.e., using technology for recreation and socialisation and not just to communicate with Home Care provider)
McWilliam^14^ et al. (2001), London	Clients living in the community aged 60–85 years., receiving home care services from a Canadian home care program clients (*n* = 6) informal care givers (*n* = 6) providers (*n* = 9)	Explore everyday experiences in home care with a focus on enactment of empowerment in the care relationship	Qualitative—Hermeneutic phenomenology, in‐depth semi‐structured interviews, audiotaped, transcribed verbatim, peer‐reviewed results	Service type: home care services Measure: experience (empowerment)	Constrained knowledge and confined authority, described as follows: degree to which participants were encouraged to pursue goals, and expectations of the system and health professionals versus pursuing goals of the participant for life and health and degree to which participants were inclined to focus on problems and limitations requiring an expert's effort to fix, rather than on strengths and resources that might be mobilised in a care partnership.
Doyle^15^ (2012), Australia	Consumers of home care package providers in Queensland, Australia (*n* = 12)	Key question: How do older people in receipt of a community care package perceive the impact of the care provided to them, including their independence, autonomy and personal fulfilment?	Qualitative, semi‐structured interview questions, face to face. Snowballing method of recruitment Heidegger's interpretive hermeneutical phenomenological approach	Service type: Home care package Measure: impact of care	maintaining personal autonomy – ‘My life is still my own’, maintaining key relationships – ‘People are the most important thing to me’, difference in power; providers exerting control over daily tasks, dictating care and quality of care, including the time‐of‐daycare is to be delivered (‘Doing battle’ and ‘Who's in charge here?’) and 3 having consistent and familiar carers who are compassionate and communicate a sense of their personal worth, cheerful and positive attitude
Day^16^ et al. (2018), Australia	Client's age (not stated), in regional NSW in receipt of home care packages from two providers (*n* = 6 completed interview, *n* = 12 completed survey, *n* = 13 completed both) Total *n* = 31	To explore experiences of older people receiving home care package support following the introduction of consumer directed care by the Australian Government in July 2015	Qualitative. Face‐to‐face interview and/or survey Convenience sampling, semi‐structured interview guide, touch point cards and emotion cards	Service type: Home care package Measure: experience (with a change of model of care)	Choice of preference; choice over constraints and choosing balance. Further analysis of consumer choice consumers feelings of being screened, consumers working out the changes (including paperwork, new accounts), consumer understanding their status: (what can I afford with the allocated funding) consumer‐seeking relatedness and relevance and consumer uncertainty about the future
Ahluwalia^17^ et al. (2010), United States	Community clients >78 years. recruited from the precipitating events project (longitudinal study) *n* = 23	To understand bathing experiences, preferences and attitudes of older persons to inform the development of effective patient‐centred interventions	Qualitative study using grounded theory framework. Semi‐structured, open‐ended interviews, audiotaped, transcribed verbatim	Service type: personal care Measure: experience	importance and personal significance of bathing to older persons; variability in attitudes, preferences and sources of bathing assistance and older persons' anticipation of and responses to bathing disability All participants placed high significance on bathing, describing it as a meaningful activity and part of an established routine
Petriwskyj^18^ et al. (2014), Australia	Clients of one large Australian Aged Care Service provider, aged 28–101 years., in 17 regions (coastal, metropolitan, rural/regional), in residential, community and retirement living *n* = 94 staff *n* = 84 clients total (43 clients in community)	To explore client engagement practices in a large Aged Care Service provider, power relations between clients and staff were examined.	Qualitative. Focus group and interviews with both clients and staff, audiotaped, transcribed verbatim. Staff (focus groups *n* = 13, interviews *n* = 9) Clients (focus groups *n* = 12, interviews *n* = 2)	Service type: home care service Measure: power relationships with care providers	Passivity, disempowerment and the bestowal of power, consumers becoming helpers and advisors to staff members and resistance, compliance and negotiation
Mahoney^19^ et al. (2019), United States	Veterans receiving home and community‐based services (*n* = 21)	To understand Veterans' opinions re: the value and impact of the Veteran‐directed home and community‐based services program on their lives	Qualitative. Focus groups (conducted via telephone), Individual interviews by telephone, semi‐structured interview guide	Service type: Home care Measure: Impact of care	Personal impact on life: ‘I'm a Person! It's a Home‐Saver’ (including physical space), how it kept them out of nursing home, allowed them to remain in a place that had meaning to them; coming back to life; ‘Keeping Me Healthy and Safe’ and 2 the importance of choice (e.g., being able to schedule their own times, choose their services and pay for services they would like).
Gill^20^ et al. (2011), Australia	Community‐based Aged Care provider in NSW, Australia. Clients >80 years. (*n* = 5), care workers (*n* = 3), managers (*n* = 1)	Key question: what factors influence the interactions of the service provider, recipients and enablers of a community‐based aged health care service, in a single service network?	Qualitative. convergent interview technique (Dick 1990)	Service type: home care Measure: Influential interactions of care	How the service is focused on the client, how the client actively contributes to the service, how the client is empowered to actively participate in the service and how the care worker is empowered to provide the care service
Gill^21^ et al. (2017), Australia	Clients >70 years (*n* = 25), Home care provider staff (*n* = 18), Informal carers (*n* = 14)	Key question: What are the shared issues and challenges being experienced by staff, their clients and informal carers with the introduction of consumer directed care?	Qualitative. Interview themes developed through iterative deductive process	Service type: home care Measure: experience	Culture: roles adopted by clients (difficulties trying to make changes, required to conform to formal rules of government and organisation) and how services were organised (no control over rostering of staff and times) Role change: client needing to direct their own service needs. Role change not supported by information that clearly explained the possibilities available to clients Operational systems: geographical coverage (unable to access preferred service provider), difficulties related to unmet needs, confusion with financial statements, third‐party contracting of services and Resourcing: personalisation of service, restrictions to timely access to resources, waiting for a particular promised service and lack of details in advance about a service
Smith‐Carrier^22^ et al. (2017), Canada	Clients >65 years. recruited across seven program sites receiving home‐based primary care (*n* = 26), academic health teams (*n* = 5) Community‐based team (*n* = 1), mobile team (*n* = 1)	To explore the experiences of patients accessing home‐based primary care (HBPC) delivered by interprofessional teams, and their perspectives on the facilitators and barriers to this model of care	Qualitative. Face‐to‐face, in‐depth interviews; open‐ended questions, inductive content analysis	Service type: home care Measure: experience	Viewed as a necessary service, promotes better patient care, improves patient satisfaction and their perception of better QoL, has challenges (personal privacy, being in a position of vulnerability) and fragmentation and disorganisation—like a patchwork of services
Fraser^23^ et al. (2014), Canada	Home care clients aged 32–86 years., Clients *n* = 1, Family caregivers *n* = 10, Total of 11 interviews and 7 pieces of artwork	Key question: What is the experience of receiving and providing home care, within the family unit?	Qualitative (arts‐based method), individual face‐to‐face interviews, interpretive description (artwork completed by client/family caregiver and stories), and convenience sample	Service type: home care Measure: client experience	Effects of caregiving: (physical, emotional, social and financial), coping with caregiving: (attitude and activities, coming to acceptance, creative problem‐solving, continuing their pattern of living), accessing home care services: (uncovering information, arranging services and equipment), working the system: (caring but limited health care system, need for advocacy), formal caregiver qualities and relationships: (capability, consistency, reliability, compassion, personality fit) and living with home care
Teale^24^ and Young, (2015), United Kingdom	Consumers of bed‐based services (*n* = 131) and home‐based or re‐ablement care services (*n* = 143)	To describe the development of Patient Reported Experience Measures suitable for use in Integrative Care services and to examine their feasibility, acceptability and scaling properties	Qualitative. Patient Reported Outcome Measures (PREMs) developed by Delphi consensus. Forty‐one of 80 questions from the 2008 Picker Adult Inpatient Survey bank were used. The final version of the PREMS was field tested in three sites (*n* = 250 service users)	Care type: Home care Measure: experience	Clients: have sufficient information, are aware of goals, are involved in goal setting, are aware of how to contact staff, questions answered, have confidence in staff, are involved in decision‐making at discharge, are given enough notice about discharge, family provided with information, requirements for additional equipment and services after discharge are discussed and are treated with dignity and respect.
Janlöv^25^ et al. (2006), Sweden	Clients aged 79 years. and older, receiving public home help (*n* = 28), who had been assessed by social workers using an assessment process aimed at identifying the clients' needs, entitlement to help, decision and follow‐up	To explore older persons' experience of participation in and influence on decisions about public home help/care when undergoing needs assessment and receiving public home help	Qualitative. A purposive sample of clients who had undergone the needs assessment process (based on the National Board of health and welfare 2002b). In‐depth interviews. Sample content analysis of conversation between interviewee and interviewer exploring the in depth meaning attached to participation in daily life	Care type: Home care Measure: experience (participation)	Assessment process (determining when help was needed) a necessary evil (balancing feelings and resources against having no choice but to accept), resources make a difference for exerting influence (having knowledge about the availability of home help and previous experience with the assessment process), feeling exposed or secure in relation to having a guardian family member present (being present but not there, ‘they were mainly talking to my wife’; family participation compensated for the power imbalance between assessors and clients. 2 Decision outcome (the actual help): incorporating home help into daily life to gain a sense of continuity, balancing daily life to gain a sense of control (what home help would come, what day, what time, how many helpers, threatened sense of personal control), balancing the relations with staff—to gain influence (having influence over the actual help provided) and power was a feature governing the interaction between the assessor and the help seeker
Norell Pejner^26^ & Brobeck (2018), Sweden	Couples aged between 65 and 80 years. receiving home care (*n* = 8) and registered nurses employed in home care (*n* = 4). Total *n* = 20	To describe how couples in need of home care services experienced receiving support from care professionals	Qualitative. Focus groups (*N* = 2; with each focus group meeting twice) and additional written diaries of everyday experiences of care were recorded Content analysis	Care type: Home care Measure: experience (receiving care—couple)	Lack of Professional Support Organisational adapted (support was inflexible and could not be adapted) Two main causes limiting (received support from several providers) and unequal access to additional support Withholding (professional not knowing client's individual situation and needs and thus withholding care) Two main causes Insufficient information and slides on responsibility (responsibility on the carer and not the provider) 2 Being in a gap (day was monotonous and routinized—focus on practical duties) Two main causes not knowing (do we need home care) and diffused (no clear rules as to who to turn to for help)
Rabiee^27^ and Glendinning (2014), United Kingdom	Older people using council‐managed personal budgets to fund care (*n* = 18)	To explore the experiences of older people who use council‐managed personal budgets (PBs) to fund home care services and their satisfaction with the level of choice and control they can exercise	Qualitative, face‐to‐face interviews	Care type: Home care Measure: experience (choice and control)	Ability to tailor home care services to personal needs and preferences was restricted to low‐level choices for older people Level of importance (choice and control): Not important choice and flexibility over agency (suggested they did not know who to choose anyway) Very important choice and flexibility over care workers, choice and flexibility over tasks (consumers reported being told what support and services were available to access, not necessarily their choice of task); choice and flexibility over timing and duration of visits (were provided with a choice and gave their choice, although often not provided) and reviews and monitoring changes (consumers could not remember being offered a review or being given the opportunity to review their support plan)
Spoorenberg^28^ et al. (2015), Netherlands	Older adults living in the community, receiving integrated care and support based on a chronic care model (*n* = 23)	To evaluate the opinions and experiences of community‐living older adults regarding integrated care and support, along with the extent to which it meets their health and social needs	Qualitative. Semi‐structured interviews, grounded theory approach	Care type: Home care Measure: experience	Experiences with ageing, ‘struggling with health’, ‘increasing dependency’, ‘decreasing social interaction’ ‘loss of control’ and ‘fears’‐ fear of falling, fear of becoming a burden to others, fear of losing independence 2 Experiences of receiving care and support from provider: ‘relationship with the case manager’—based on equality and confidentiality; ‘interactions’‐ with providers seen as supported, monitored, informed and encouraged and ‘feeling in control, safe and secure’—support, monitoring, information and encouragement participants received helped them to feel in control, safe and secure
Wilde^29^ & Glendinning (2012), United Kingdom	Older people using re‐ablement services (*n* = 34) and carers (*n* = 10)	To explore the perceptions and experience of home care users of re‐ablement services	Qualitative, semi‐structured interviews, thematic analysis	Care Type: Home care (re‐ablement service) Measure: experience	Participant confusion over service aim, large numbers of care staff making contact to set up service (limited by capacity and understanding in some instances), written information provided was overwhelming and unclear, participant expectation of home care (not understanding re‐ablement, presuming tasks would be done for them), not all participants felt involved in goal setting—feelings that the service was not open to negotiation, difficulties understanding a goal‐orientated approach (for participants with progressive conditions), frustration with limited access to additional resources (such as physiotherapy) to assist with re‐ablement focus and some participants refused to participate (not compatible with cultural preferences)
Simons^30^ et al. (2016), Australia	Older people receiving home care packages from Brotherhood St Lawrence (BSL) home and community care services (*n* = 45)	To evaluate the efficacy and sustainability of the BSL Consumer Directed Care model and to contribute to broader debates about the individualisation of aged care	Qualitative. Consumer interviews for existing clients (*n* = 45), consumer forums (existing and potential clients) (consumers *n* = 99 and carers *n* = 24). Two focus groups (both focus group consumers *n* = 5 and carers *n* = 2)	Care type: Home care Measure: experience	Consumer confusion: not understanding Consumer Directed Care (too much information—overwhelming and confusing), level of autonomy (between consumers and case managers) and number of allocated visits for case managers Consumers satisfied: introduction of the monthly budget statement, however, found them difficult to interpret and understand and flexibility and control—spending funds in line with their health and well‐being goals
Tiilikainen^31^ et al. (2019), Finland	Older people, living alone, receiving social care services (*n* = 19)	To explore older person perceptions of quality of life from the perspective of access and use of health and social care services	Qualitative. Focus group, recruited via local health and social care services Interview based on four domains of WHOQOL‐BREF everyday social relations and knowledge of services, continuity in life, recognition and appreciation of older people and access and use of services Thematic analysis of focus group discussion.	Service type: Home care Measure: Client perception of services on quality of life	Access to and use of social services: bouncing around from service to service without getting help, professionals seen as gatekeepers or ‘brakes’ inside the system, clients felt that their concerns often ignored, deciding at what point to ask for help, Worried about others with ‘poorer health’ than themselves who did not have resources either (prioritising others first) and clients with no children found access to services more difficult. Recognition inside the services: dissatisfaction with the amount and type of home care—(limited staff time, high staff turnover) and mistreated by professional staff and being seen as a burden (care staff asking, ‘what do I have to do’ ‘I do not have much time’) 2 Access to Information information rarely provided even if you were eligible for services Quality of life—what is important to clients Accessing health and social care was closely linked to autonomy in everyday life Adequate services to maintain meaningful hobbies and social bonds and to participate in community Ability to access information about existing services (needed someone to advocate for them, who knew the system)
Russell^32^ et al. (2020), Australia	Older people receiving a home care package (*n* = 37), from urban, regional and rural Australia. Age range 66–95 years. The sample included minority groups including ATSI (*n* = 1)	To explore consumers' experiences of receiving a home care package in Australia	Qualitative study, face‐to‐face or phone interviews using an interview schedule (9 key questions)	Care Type: Home care Measure: experience	Factors important to older people receiving home care packages: (1) access to reliable information; (2) providers who deliver high‐quality consumer directed services; (3) being charged reasonable fees (for equipment, support workers and case management); (3) case management delivered by staff who are experienced, qualified and easy to contact; (4) support workers who are suitably trained, competent, trustworthy, punctual and empathetic; (5) to be involved in decision‐making, listened to and heard (person‐centred care) and (6) to have access to social activities and community life
Fæø^33^ et al. (2020), Norway	Older people (>65 years) with dementia, living at home (*n* = 12). Six received home care service; all attended daycare services. Age range 69–89 years	To explore the perceptions of older people living with dementia on assistive technology, volunteer support, home care services and daycare centres	Qualitative, exploratory design, based upon hermeneutical methodology. Semi‐structured interview questions (experiences of, and attitudes to receiving support)	Care Type: Home care Measure: Perception	Care and support experienced is balanced between being seen as supportive versus offensive and being seen as dignity preserving versus violating This perception is based on individual preferences, and how they perceive the care and support that might impact their daily life
Compton^34^ et al. (2020), Canada	Older people living at home (>75 years.) receiving Home First services (*n* = 8 consumers, *n* = 11 caregivers)	To explore older people's perceptions of community‐based services delivered by Home Care Home First	Qualitative study—interpretive descriptive design. Semi‐structured interviews	Care Type: Home care Measure: Perceptions of services	Four themes: Growing older in chosen place with support (the importance of being able to stay at home) Philosophy of care (holistic, client‐centred, positive relationships, caring and compassionate staff) Processes (positive: adapted to individual needs, negative: not delivered at appropriate times) Significance of Home First for clients and their family caregivers (accessible—i.e., available, but only when you know they exist)
Wennman^35^ et al. (2020), Sweden	Oncology patients living at home and close relatives, age range 53–87 years (patients *n* = 11)	To explore patients affected by life‐threatening illness and their families' experience of receiving home care	Exploratory qualitative study. Face‐to‐face, unstructured in‐depth interviews	Care Type: Home care Measure: Experience of care	Three themes: Create a safe environment (respond to significant needs such as pain, communication, psychosocial support; involve the person in decision‐making processes) See the person (as a person with individual needs and not just a patient) Better to manage care at home (relief to be treated at home, hospital was seen as physically and psychologically challenging)
Askew^36^ et al. (2016), Australia	ATSI people living in the community, with a chronic condition, regularly attend the local health service Quantitative measures obtained (*n* = 37); qualitative interviews with patients (*n* = 9), spouse (*n* = 1) and staff (*n* = 15)	Feasibility, acceptability and appropriateness of chronic disease management using a case management approach integrated within an urban ATSI primary healthcare service	Qualitative study—In‐depth interviews, Quantitative—data from patient records (baseline, 3 and 6 months)	Care Type: Case management in the community Measure: Experience and acceptance of case management	Results (qualitative interviews) Case management model universally acceptable Patients appreciated home visits from case managers in their own homes (provided an increased sense of safety), case managers showing interest in their lives, providing holistic care and removing daily stressors Mutual respect in the relationship, and cultural acceptance was seen as a positive

Abbreviations: ATSI, Aboriginal and Torres Strait Islander; QOL, Quality of Life; WHOQOL—BREF, world health organisation quality of life—Brief version.

**TABLE 5:** Key methodological details and results of included studies measuring access to and choice of services. (*n* = 3)

Study: Author, Country	Population	Aims	Methods	Key measure & service type	Key findings
Council on the ageing Australia^32^, [COTA] (Feb 2018)	Consumers receiving aged care services in the community (*n* = 30), and providers delivering services (*n* = 64)	To consider systems and indications for quality and safety in aged care. Key question: what do consumers think is most relevant when choosing an aged care provider?	Online survey developed via Qualitative plug‐in methodology with consumers in South Australia (*n* = 67) and focus groups (invited: *n* = 65 consumers, *n* = 93 providers; attended *n* = 30 consumers, *n* = 64 providers) Online survey completed (*n* = 676, consumers and representatives, *n* = 416 providers)	Service type: Home care services Measure: choice (services)	*Consumer experience metrics*: Consumers want more information about consumer experiences with services *Quality of life metrics*: Consumers ranked the following QOL indicators as important: being treated with respect and dignity, staff friendliness, feeling safe and secure, being supported and encouraged to raise concerns with the service, a sense of independence, control over daily life, being supported to maintain social relationships and connections with the community, maintaining and supporting spiritual, cultural, sexual and religious identity
Sefcik^33^ et al. (2016), United States	Hospital inpatients aged >55 years. (*n* = 50) Inpatients (surgical and medical wards in a large metropolitan hospital)	Key question: What do hospital patients want to know about post‐acute services to help them make an informed decision?	Qualitative descriptive conversational content analysis Interviews audiotaped and transcribed verbatim	Service type: Post‐acute care Measure: choice (information)	Patients want: Practical information about services (cost, extent of the service, quality rating and staff qualification), to understand ‘how it relates to me’ (service expectations of them, personal benefits they would receive, how services related to their own condition) and opportunities to understand Post‐Acute Care (receiving clear information [written and verbal]).
Suurmond^34^ et al. (2016), Amsterdam	Older people (> 50 years) living in low socio‐economic neighbourhoods in Amsterdam receive home care services. (group interviews *n* = 50; individual interviews *n* = 5)	To explore barriers to access home care services of Turkish Moroccan Surinamese and ethnic Dutch elderly	Qualitative. Group interviews (*n* = 50) and Individual interviews (*n* = 5) Experiences and perceptions on navigation and access to services Two‐stage analytic process: coding based on content and themes mapped against categories of Levesque framework	Care type: Home care Measure: Barriers to access	Barriers to accessing home care identified: Perceiving a need for home care: lack of knowledge (about functional limitation), preference for family members to provide home care. 2 Searching for home care: lack of knowledge (about how to search, where to search and what entitled to) and unmet expectations (did not know they could discuss home care with GP, waited for GP to discuss with them, GP assumed family would provide care for ethnic minority). 3 Engaging with home care: inability to engage—language and communication difficulties

**TABLE 6:** Key methodological details and results of included studies measuring client's preferences and expectations of care (*n* = 14)

Study: Author, Country	Population	Aims	Methods	Key measure & service type	Key findings
Chester^35^ et al. (2018), United Kingdom	Participants with dementia attending memory clinics and a carer organisation. Participants with dementia (*n* = 44) carers (*n* = 103)	To investigate people with dementia and their caregivers' preferences for home support services in early stage dementia	Discrete choice experiment survey developed by a systematic review of evidence and consultation with patients (*n* = 2) and public reference groups (*n* = 2) Face‐to‐face interviews (*n* = 104 including all 44 with dementia) and online questionnaires (*n* = 43) Totally, 147 participants completed the discrete choice experiment	Service type: memory clinics and carer organisation Measure: preferred service attributes	Most preferred attributes (consumers): opportunities for social and recreational activities (provided by a dedicated worker at home) and support with feelings and concerns Most preferred attributes (carers): support with personal feelings and concerns (provided by a trained counsellor at home) and information on coping with dementia (provided by an experienced worker at home)
Kaambwa^36^ et al. (2015), Australia	Consumers of five aged care providers in Australia (>65 years.) receiving home care *n* = 87 consumers, *n* = 30 carers	To investigate what consumers of home care service preferences are on important features of consumer directed care	Discrete choice experiment (two home care packages developed from a previous qualitative study involving consumers). Consumers voted on the preferred package	Service type: Home care package Measure: preference	Ability to save all or half of unused funds from their community aged care package for future use, ability to choose some of the workers that provide their day‐to‐day services and ability to have fully flexible support workers and change activities within the care plan
Harrison^37^ et al., (2014), Australia	Clients >65 years. living in the community, approved for government‐funded community aged care services (*n* = 55), clients with participating carers (*n* = 37)	To investigate the relationship between clinically assessed care needs and expectations of older people and their carers	Cross‐sectional research, baseline CENSUS data. Aged Care Assessment was used to assess clinical needs and identify met and unmet needs Satisfaction with care was assessed using a modified 22‐item questionnaire devised for carers of stroke patients	Service type: Home care Measure: Assessed care needs and expectations of care	Two needs were assessed as being met: mobility and home maintenance (shopping, errands, housework and laundry). Eight needs were assessed as being unmet: hearing; pain; skin care; breathing; nutrition (weight and eating); social behaviour (pursuit of social interests, socialising, maintaining relationships); mental health and self‐care/toileting Strong correlation between more unmet needs and lower QOL, higher cognitive impairment and more carer burden Clients' expectations of care: Domestic support, personal care, transport and shopping were the most common expectations of clients
Abbott^38^ et al. (2018), United States	Nursing home clients (*n* = 255), Older adults receiving home and community‐based services (*n* = 528), mean age of clients 76 years	To identify the top 10 shared preferences that are important to most consumers receiving long‐term services and supports	Cross‐sectional survey design, preference assessment interviews using the 55‐item PELI (HC)—(Preferences for everyday living inventory—home care)	Service type: home care Measure: preference for care	Top 10 preferences (home care): Keeping in contact with family, spending time outside, having certain family or friends involved in your life, privacy, music, keeping things in a certain place, giving gifts; being active at certain times of day and choosing what to eat and travelling
McCaffrey^39^ et al. (2015), Australia	Older people aged >65 years. receiving home‐based services from five providers in two Australian States (*n* = 17) and informal carers >18 years (*n* = 10)	To determine what attributes of consumer directed, home‐based support services are important to older people and their informal carers to inform the design of a discrete choice experiment	Qualitative. Semi‐structured face‐to‐face interviews, question topic guide developed from the literature review Thematic and constant comparative analyses	Care type: Home care Measure: preference (service characteristics)	Six attributes of service characteristics choice of providers: services can be provided by a single provider, multiple providers or multiple providers plus others choice of supporting workers (including choice of informal support workers) flexibility in care activities provided: the support worker can change activities on the care plan as directed by the individual contact with the service co‐ordinators: high, medium or low face‐to‐face contact managing the budget: the budget is managed by the individual, informal carers or service providers and saving unspent funds: all, half or none of the unused funds can be saved
Moran^40^ et al., (2012), United Kingdom	Older people in the individual budget pilot project (qualitative interviews *n* = 40) and quantitative analysis of older people's outcome measures (*n* = 263)	To assess the impact and outcomes of older people participating in the English Individual Budget (IB) pilot projects	Qualitative, multimethod evaluation. Consumers randomised into two groups Standard outcome measures (*n* = 263) and qualitative semi‐structured interviews (*n* = 40)	Care type: Home care Measure: preference (budget)	Preferences: First preference: receive the budget in cash Second preference: budget managed by the local authority Third preference: payments paid into a joint bank account held by the client and another person Least popular preference: budget administered through a third party Budget use: Majority of older people used their budget to purchase conventional mainstream services (including home care, meals, equipment and adaptations, accommodation, short breaks and transport) and personal assistance
Low^41^ et al. (2013), Australia	Consumers living with dementia, receiving aged care services, aged between 30 and 86 years. (person living with dementia *n* = 1, carers *n* = 31); Providers of aged care services (*n* = 32) and policy representatives (*n* = 4)	To examine the views of consumers, service providers and policy representatives regarding important characteristics and outcomes for community care (for persons with dementia)	Qualitative. Face‐to‐face, telephone or group interviews, open‐ended questions. Thematic analysis	Care type: Home care Measure: Characteristics of home care (for persons with dementia)	Desired outcomes for persons with dementia: personalise activities—provide stimulation and enjoyment and be comparable to likes and interests increased socialisation (with or without carers) outside the home environment provide for a routine in daily life maintain quality of Life and personalised activity participation and socialisation Desirable community care services for person with dementia carer/client education, carer support group, daycare/respite, emergency care and help with daily living skills and transport Desirable characteristics of care staff delivering care: flexible, ability to engage, enjoy their job, treat with respect, dementia trained and personality appropriate Issues relating to the aged care system: accessibility, consistency of care across services, flexibility (lack of), standard of care and insufficient funding
Laragy and Vasiliads^42^ (2020), Australia	Older people receiving home care packages from seven Australian aged care providers. Consumers and informal carers (*n* = 99). Age range; consumers (53–100 years.) and informal carers (38–97 years.)	To understand the expectations of older people who participated in a self‐managing home care package trial	Quantitative and Qualitative study. Baseline Questionnaire (Quantitative analysis: *n* = 99) and selected follow‐up semi‐structured interviews (qualitative analysis: *n* = 18). Three areas examined: motivation, expected outcomes and attitude towards risk	Care Type: Home care Measure: Expectations of self‐managing home care package	Older people's expectations of self‐management options (qualitative data): provide increased choice and control provide greater flexibility to use funds reduce administration fees charged by aged care providers more funds to spend on services and supports of choice improved relationships with service providers and opportunities to choose their support staff Older people perceived risks of self‐management options (qualitative data): run out of funds spending funds inappropriately compromise clinical care employ unsuitable or unqualified staff and possible poor outcomes
Sanerma^43^ et al. (2020), Finland	Older people living at home receiving home care services (>70 years.). Age range 70–96 years. Six families recruited (*n* = 6) older people and (*n* = 7) family members, total (*n* = 13)	To evaluate client‐centred care in home care services from the perspectives of older adults	Qualitative study. Interview questions: content and services of home care, assessment and practice of home care and development of client‐centredness in home care	Care Type: Home care Measure: Perspective and preference	An individual's life situation influences service needs and service experience. Services—getting services was the most difficult; need to be available when required, trustworthy and delivered in collaboration with the older person and family members. Staff—professional competence, high level of interpersonal skills, flexible, reliable, mutual respect, a good listener and trustworthy. Choice of services provided, to participate in activities. Overall: Client‐centred home care services must be based on the older person's individual life context and realised by the social mechanisms of: interaction, participation, adaptation and trust
Wang^44^ et al. (2022), Hong Kong	Older people living in the community (> 65 years., Cantonese speaking) who used community‐based long‐term care services. Excluded: people with severe dementia, mental illness and cognitive impairment	To extract older community care service users' preference of community‐based long‐term care, on of flexibility in service provision	Cross‐sectional survey with a Discrete Choice Experiment (DCE). Focus group (*n* = 25, including older people) informed DCE attributes). Cross‐sectional survey with DCE (*n* = 318). Generalised multinomial logistic model applied to determine the relative importance and willingness to pay attributes	Care Type: Community care Measure: Preference and willingness to pay	Willing to pay higher prices for community services that are flexible and have more frequent meetings with case managers and social workers Prefer to receive information and be supported to make decisions by social workers Preference for having greater choice and control of services provided
Holloway^45^ et al. (2015), Australia	Key stakeholders across metropolitan, regional and rural Australia (total *n* = 172). Consumers represented (*n* = 17), >65 years. or >45 years. for ATSI	To gain the perspectives of keyholders to inform the development of the Australian national guidelines for providing palliative care in community aged care settings	Descriptive exploratory qualitative study. Semi‐structured individual interviews and focus groups	Care Type: Home care Measure: Perspectives of care	Palliative care approach Need to recognise the difference between a palliative care approach and specialist palliative care. A gap exists between aged care services and specialist palliative care Carer support Involve caregivers in decision‐making, consider both client and carers wishes Advance care planning Seen as invaluable, but infrequently used and poorly developed. Need for advanced care plans to be developed early Physical and psychological symptom assessment and management Difficulties associated with assessments being conducted by multiple care providers, limited or no access to a common database, lack of consistent care staff and use of locum GPs Psychosocial support Imperative to be able to remain at home, but concern over invasion of the home environment and the creation of a clinical environment in the home Spiritual support Early assessment of spiritual needs is essential. Spiritual is viewed as greater than just religious needs. Diverse cultural and language groups Careful assessment and cultural sensitivity are required. Communication barriers noted. Special need groups Importance of equitable home care opportunities, particularly for those who live alone and are without family support
Swain & Barclay^46^ (2015), Australia	ATSI people across Australia, living in the community (*n* = 102); approximately 90% were >40 years. 23 were previous users of the Home medicines review program	To explore ATSI perspectives on the Home Medicine Review Program	Qualitative, semi‐structured focus groups. An ATSI Advisory group was established to guide the design and data collection phase	Care Type: Community service Measure: Perspectives on receiving the service	Overall: Desire to better understand their medicines Useful service, but process confusing and confronting (very little information provided about the service) The process needs to be managed in a culturally accepted way Two themes: Cultural considerations: service must be organised by their local ATSI health service, pharmacists must have a relationship with the health service, given the option for the service to be conducted at home or at the health service, prefer to have a health worker from the health service present at the medication review, and involve family members Adapting to meet the needs of ATSI clients: Explain the process beforehand; information sheets are a good option; ATSI health workers/nurses should be able to refer for the service, preference for specialist aboriginal health workers in medication review and management.
Angell^47^ et al. (2018), Australia	ATSI people >40 years. (*n* = 60), participating in an evidence‐based culturally appropriate falls prevention program	To examine preferences and willingness to pay of consumers, for participating in a fall prevention class, and the importance of transport, cost and cultural appropriateness in choice	DCE (face‐to‐face survey) assesses three attributes (out‐of‐pocket expenses, transport options and culture‐specific delivery). Quantitative analysis	Care Type: Community health program Measure: Client preference	Out‐of‐pocket costs and whether the class was culturally specific were high predictors of choice
Lukaszyk^48^ et al. (2017), Australia	ATSI people >45 years. living in the community, attending aboriginal health and/or receiving community services (*n* = 76)	To understand Older ATSI peoples' perceptions and beliefs about falls and to identify desired elements of a fall prevention program	Qualitative study. Yarning circles (*n* = 10), semi‐structured topic guide; conventional content analysis; results reviewed by a senior Aboriginal Community spokesperson	Care Type Community health program Measure: Perceptions and preference	Impact of falls: Physical disability (temporary and permanent), rehabilitation services inflexible, too costly and difficult to access Loss of emotional well‐being (loss of confidence, frustration and anger, depression, fear and embarrassment) Loss of connection to family and community (isolation from community, guilt from relying on others and loss of family responsibilities) Fall prevention: Use of existing fall prevention programs (unaware, not available and no referral provided) Knowledge (past experiences and willingness to share with others) Interest (want more information about the impact of ageing on falls) Preferred attributes of the program: Type (aboriginal specific and meet the needs of that community) Delivery (group‐based, flexible, long‐term and run through Aboriginal organisations) Accessibility (gold coin donation, transport provided)

**TABLE 7:** Key methodological details and results of included studies measuring service quality (*n* = 6)

Author	Population	Aims	Methods	Key measure and service type	Key findings
Jones^42^ et al. (2007), England	Thirty‐four local authorities in England provide home care; random sample selected >65 years. accessing services in 2003 (*n* = 21,350)	Key questions: Q1. Do the performance indicators reflect home care quality? Q2. What are the underlying constructs of home care quality?	Qualitative. Self‐completed questionnaires (by phone and face to face [when necessary]) *n* = 8700 service users *n* = 21,350 (completed extended questionnaire)	Service type: Home care services Measure: service quality	Two performance indicators that reflect quality: satisfaction of consumers with service and suitable times for delivery of services. Underlying constructs of home care quality satisfaction and suitable times
Firbank^43^ (2012), Canada	Four Home care agencies implementing quality improvement programs *n* = 33 clients *n* = 13 carers *n* = 39 front‐line workers	To assess different instruments used to gather information on home care service quality from consumers and challenges encountered when trying to prioritise their views	Qualitative. In‐depth, face‐to‐face interviews with flow charts were used to describe the process Consumers were asked to identify, and rank order, three quality issues most critical to them	Service Type: Home care services Measure: service quality	Two measures of poor quality: care relational aspects (poor professional attitude and communication) and process‐related or structural aspects (service continuity and work organisation, i.e. restricted service hours, confirmation of service changes, professional boundaries) and insufficient service volume
Gregory^44^ et al. (2018), Australia	Home and community care Older people (*n* = 7) Carers (*n* = 8) Key informants (people with expert knowledge about services to support older people at home, *n* = 11)	To gain an in‐depth understanding of what is involved in providing good quality healthcare to older people who need support to live at home	Qualitative. Semi‐structured interviews, interview guides, and interpretive descriptive approach	Service type: Home care Measure: quality of care	Partnerships in healthcare being treated as an equal, being involved in decision‐making and making contributions which impact healthcare and health systems. invisibility of older person as a partner in healthcare: older person not seen as a partner in care, older person not placed at the centre of care as a partner in care and older person is more visible as a recipient of care and is not yet placed at the centre of healthcare as a partner in care
Samuelsson^45^ & Wister, (2000), Canada	Clients aged 64–97 years. receiving home care services *n* = 76	To explore clients' expectations of quality in home care services and their perceived satisfaction with services	Qualitative; face‐to‐face interviews. MAUT scaling procedure (measures relative importance assigned to the value of consumers home environment) Organises quality attributes into a tree diagram ‐ this study expanded this design to include social relationships	Service type: home care Measure: quality and satisfaction	Quality attributes (suitability of home care): Highest ranked personal qualities of carers (good at giving care, pleasant and friendly, respectful and considerate) and professional competence of carers (high‐skill level) Lowest ranked personal relations (sympathetic) and time (plenty of time, and times are kept). Satisfaction: Highest ranked personality characteristics, such as ‘home care giver is honest and reliable’, ‘home care giver is pleasant and friendly’, ‘home care giver is quiet and reliable’ and ‘home care giver is respectful and considerate’ Lowest ranked time/availability issues, such as ‘times are kept’, ‘the times at which you receive help’ and ‘There is plenty of time to do the work’.
Cohen‐Mansfield^46^ et al. (2018), Israel	Households with clients >60 years, employing migrant live‐in carers in Tel‐Aviv and Jerusalem Clients *n* = 72; relatives providing care *n* = 117	To establish the components of quality of care as provided by migrant live‐in caregivers. To explore underlying dimensions of quality of care to clarify its meaning in the context of in‐home care of frail older persons	Qualitative. Twenty‐three demographics analysed using descriptive statistics. Quantitative and qualitative analyses. Quality of Care Questionnaire (QuCQ) was developed for this study	Service type: home care Measure: quality of care	Six components of Quality of Care support provided in needed areas caregiver answering persons' needs caregiver promptness in answering needs, understanding one another, caregiver knowing the older person as a person, caregiver knowing older persons main concerns and caregiver treats older person with respect Quality of care: (Older Person) ‘caretaking’—answering older persons' needs and treating them with respect and ‘relationships’—knowing the older person as a person, knowing the main concern, and understanding one another. Quality of care (Relatives): ‘caretaking’—answering older persons' needs and ‘relationship’—knowing the older person and knowing the older persons' concerns Analysis: interpersonal aspects of care are contributed to older persons' quality of care. Quality of care is dependent on 1: caregivers' receptiveness to physical care needs and 2: caregivers' ability to interact with older persons and understand them beyond ‘surface’ care needs
Groenewoud^47^ et al. (2008), Netherlands	Semi‐structured interviews (*n* = 22 [12 experts in home care and 10 experts in residential aged care]); Home care focus group (consumers *n* = 13; experts in home care quality *n* = 14)	To identify ‘building blocks’ for quality report cards for geriatric care	Qualitative Consumer focus groups Open brainstorming on quality aspects is considered important when choosing providers. Prioritisation of the building blocks generated from the expert panel; concept mapping/structure conceptualisation used to define building blocks	Service type: Home care Measure: quality of care	Overall summary: home care consumers attach more value to the availability, continuity and reliability of care (i.e. receiving high‐quality care from qualified healthcare workers at appointed times) and consumers want information on structure, process and outcome indicators and rating outcome indicators such as effectiveness and safety of care. Eight home care clusters (quality): availability, continuity, reliability and organisation of care; waiting time; staff expertise and effectiveness of care safety; personal care plan; privacy, respect and autonomy; complaints; participation and choice; informal care and Information

### 3.2 Identifying themes

The literature revealed recurring patterns of client responses, in each of the key outcome measure categories adopted by each study. This resulted in the development of definitions of key measures of quality, from the client's perspective.

#### 3.2.1 Measures of quality

This stage identified five key quality measures that were most frequently examined in the included articles: (1) client satisfaction, (2) client experience, (3) access and choice, (4) preference and expectations of care and (5) service quality. Definitions of these measures are summarised in Table 2, and the characteristics of the included studies according to each of these quality measures are summarised in Tables 3, 4, 5, 6, 7.

Of the included articles (39%; *n* = 24) explored client's perceptions and experiences and (24%; *n* = 15) explored satisfaction with the services they received. Four of the articles focused on other topics including understanding the needs and preferences of clients when seeking information about community services (Sefcik et al., 2016), investigating the relationship between clinically assessed care needs and the expectations of older people and their needs (Harrison et al., 2014), the enactment of empowerment in the care relationship experienced by the older person (McWilliam et al., 2001) and the impact and outcomes of older people in light of financial system changes in England (Moran et al., 2012). One article investigated clients' perspectives on the impact of the care and services they received (Doyle, 2012), and one examined consumer satisfaction in relation to workforce issues (Anderson et al., 2006). One article explored client's satisfaction against the process and structure‐related factors (Kajonius & Kazemi, 2016). Four articles focused on clients from culturally and linguistically diverse backgrounds and explored their experiences and satisfaction with aged care services (Aletras et al., 2010; Kwak et al., 2017; Skaperdas et al., 2010; Suurmond et al., 2016). Five articles specifically included Aboriginal and Torres Strait Islander clients and explored their experiences of chronic disease management using a case management approach (Askew et al., 2016), their perceptions of palliative care (Holloway et al., 2015), their perspectives of the Home Medicine Review Program (Swain & Barclay, 2015) and their preferences of participation in a fall prevention program (Angell et al., 2018) and desired elements of a fall prevention program (Lukaszyk et al., 2017).

### 3.3 Drawing conclusions from the data

Results of step 4 of the analysis identified five quality domains, within the five key measures of quality that were used to obtain the results of the included articles. A summary of the key attributes of each quality domain, as viewed by clients, is presented in Table 8.

**TABLE 8:** Quality domains developed from the synthesis of this literature

Quality Domain	Identified by key quality measures used in included studies*	Key attributes—client's views
Staff knowledge	Client satisfaction^a^, client experience^b^, access and choice^c^, preference and expectation of care^d^ and service quality^e^	Receiving care and support from competent and knowledgeable staff^a^, consistent and appropriate care that meets client's individual needs^b^, support and information to understand how care relates to individual clients^c^, being informed of the different types of care available^c^, having staff who are well trained and knowledgeable^d^, professional staff who treat clients with dignity and respect^e^, highly trained staff^e^
Respect for clients	Client satisfaction^a^, client experience^b^, access and choice^c^, preference and expectation of care^d^ and service quality^e^	Being treated with dignity and respect^a^, being treated as an equal person in the care relationship^a^, equality in the care relationship^b^, consistent and appropriate care that meets their individual needs^b^, being empowered to contribute to their own care^b^, being treated with dignity and respect^c^, having choice and control over daily life (including the ability to maintain social connections with their community, spirituality, cultural, sexual and religious identity)^c^, services delivered by professional staff who treat them with dignity and respect^e.^
Person centred approach	Client satisfaction^a^, client experience^b^, access and choice^c^, preference and expectation of care^d^ and service quality^e^.	Valuing the importance of being able to make decisions about the care they received^a^, being provided with the opportunity to maintain their personal autonomy^b^, having choice over the care provided^b^, having the opportunity to participate in their own care (inclusive of those with language and communication difficulties)^c^, having flexibility to change care plans in accordance with individual preferences^d^, having opportunities for connectedness and engage in social and recreational activities^d^, having the choice of support workers who provide care^4^, choice over what care types are delivered with funds allocated^d^, having suitable times for the delivery of services^e^, having care that meets their needs and is provided by staff who see them as a person beyond their care needs^e^ and care that is personalised and adapted to their individual preferences and needs^d^
Collaborative partnership with a client	Client satisfaction^a^, client experience^b^, access and choice^c^, preference and expectation of care^d^ and service quality^e^	Having positive interpersonal relationships with staff^a^, being provided with sufficient time to talk to staff^a^, being treated as an equal partner in the care relationship and being involved in the decision‐making process^e^
Clear communication	Client satisfaction^a^, client experience^b^, access and choice^c^, preference and expectation of care^d^ and service quality^e^	Being provided with information that is easily understood to enable informed decisions about care^b^, receiving appropriate information to inform choice (including consumer experience measures)^c^, being supported and having information to understand how care related to them^c^, wanting to be informed of the different types of care available^c^

* see Table 2.

#### 3.3.1 Quality domain 1: Knowledge of staff

It was evident from the literature that clients were more satisfied when receiving care and information from staff who were perceived to have high levels of knowledge. Clients highly value staff being knowledgeable about the service(s) they provide(ed), the way in which care should be delivered, and understanding clients' individual circumstances and problems (Aletras et al., 2010; Mason et al., 2004; Teale & Young, 2015). Knowledge was also viewed as important when providing information to clients about the services and care they were receiving. This included practical aspects such as the cost, extent and duration of the service, quality rating and the qualifications of the staff providing the care (Sefcik et al., 2016). It was important to clients that staff use their knowledge to inform them of their responsibilities in the care relationship, including how the service related to the client and their identified needs, what the service expected of them and what benefits they would receive from the service (Sefcik et al., 2016). The skills and competence of staff consistently affected a clients' satisfaction rating. Anderson et al. (2006) reported clients valued having well‐trained, highly knowledgeable and competent staff just as highly as they rated having sufficient staff to provide the services. Clients valued a highly skilled workforce who received adequate training to meet their individual needs (Anderson et al., 2006; Royal Commission, 2020; Spoorenberg et al., 2015). The relationship between a highly skilled workforce and client satisfaction was also evident with clients valuing the use of skilled social work professionals to act as care managers coordinating a clients' care needs (Chesterman et al., 2001; Wang et al., 2022). Having consistent staff who were well trained was a strong theme throughout the literature that resulted in improved client satisfaction (Byrne et al., 2011; Groenewoud et al., 2008; McCaffrey et al., 2015; Royal Commission, 2020; Samuelsson & Wister, 2000). Lack of professional support provided to clients was identified as another contributing factor in the knowledge of staff quality domain. For instance, Norell Pejner and Brobeck (2018) found clients reported a lack of professional support when seeking out services, which, from the clients' perspective was viewed as professionals lacking knowledge about their individual care needs and situation, and thus withholding care. One particular study explored the differences between staff and clients' perceptions of clients' psychological well‐being and found clients rated staff knowledge and their ability to motivate them to engage in activities outside the home, engage with community groups within their age group and engage in hobbies of their interests, as very low (Eloranta et al., 2010). This study reflects the emphasis placed by clients on the importance of community services focussing on the overall psychological well‐being of clients.

#### 3.3.2 Quality domain 2: Respect for client

Treating a client with dignity and respect was an underlying theme that featured strongly in most studies. Two studies focussed on clients' culture and religious preferences, identifying the high‐value clients placed on the cultural and religious competence of the provider, enabling staff to treat them with dignity and respect (COTA, 2018; Mason et al., 2004). One study identified specific cultural considerations for Aboriginal and Torres Strait Islander people and the importance of providing community services that are organised by their local health centre in preference to an aged care provider, promoting a trusting and respectful relationship (Swain & Barclay, 2015). Being treated with dignity and respect was rated by clients as a significant indicator contributing to their quality of life, and in home care, quality of life was viewed as more important to clients compared with the quality of care (COTA, 2018). Clients described wanting their privacy respected and being treated with dignity as an important service experience that would increase their likelihood of choosing a provider (Bikker & Thompson, 2006). Clients described common failures of a home care service relating to process or structural aspects, including professional boundaries, describing poor professional attitudes resulting in inappropriate communication between staff (Firbank, 2012). Kajonius and Kazemi (2016) also found process structures were highly important to clients when receiving home care and included being treated with respect, having influence over decisions and receiving appropriate information. A strong and consistent indicator of client dissatisfaction in the study by Anderson et al. (2006), included the mistreatment of clients by staff, with clients insisting staff treat them with dignity and respect. The Australian Royal Commission on aged care quality and safety (2021) explored the quality of care, quality of life and concerns and complaints of aged care clients. Concerns identified by clients as being of high importance included clients being treated like a child, staff body language and staff attitude or conduct which was perceived as staff being rude, unkind and unsupportive. Clients also reported reasons for their unwillingness to share their concerns with others, including not knowing to whom to report their concerns, not knowing how to report their concerns, not having the capacity to report or fearing repercussions if they reported a concern. Samuelsson and Wister (2000) investigated clients' expectations of quality in home care services and found suitability of home care, described by clients as the personal qualities and professional competence of staff, to be the most important attribute. Teale and Young (2015) involved clients in developing a patient‐reported experience measure (PREM) for use in integrative care services. Their findings identified 11 themes that contributed to the development of the PREM, including clients wanting to be treated with dignity and respect. Low et al. (2013) specifically looked at outcomes of community care for persons with dementia and found one of the most desirable characteristics of care was treating the client with dignity and respect.

#### 3.3.3 Quality domain 3: Person‐centred approach

Choice and control for clients were seen as major contributor to satisfaction increasing their quality of life. Being able to choose what services a client desired, nominate a preference for the timing of those services to be delivered, and being involved in the review of their effectiveness were strongly represented in the literature (Aletras et al., 2010; Bikker & Thompson, 2006; Byrne et al., 2011; COTA, 2018; Gill et al., 2017; Jones et al., 2007; Kaambwa et al., 2015; Mason et al., 2004; McCaffrey et al., 2015; Rabiee & Glendinning, 2014; Royal Commission, 2020; Samuelsson & Wister, 2000; Skaperdas et al., 2010). Not only did clients want providers to understand their basic physical needs, but they also wanted them to recognise their need to maintain meaningful hobbies and social bonds, participate in the community and be given a choice about what social interaction they desired (Royal Commission, 2020; Tiilikainen et al., 2019). Clients described impacts of care they did not like such as care that became a list of tasks performed by strangers, with whom they had no personal relationship, care that did not accommodate their preferences regarding how things should be done (Doyle, 2012), care that forced them to do things they did not like (Aletras et al., 2010) and care that required them to conform to the formal rules of both governments and organisations in terms of what, when and how care was to be delivered (Gill et al., 2017). Cohen‐Mansfield et al. (2018) explored the underlying dimensions of quality of care and found interpersonal aspects of care are significant contributors to an older persons' quality of care rating. This included knowing the older person as a person, knowing their main concerns and mutual understanding between care staff and the older person. Gregory et al. (2018) identified the views of clients wanting to make contributions to their care but were more visible only as a recipient of care and not placed at the centre of care or contributing as a partner in care.

#### 3.3.4 Quality domain 4: Collaborative partnership with clients

This quality domain emerged from a deeper exploration of the literature. While it is recognised that engaging with clients and sharing the decision‐making process is a component of person‐centred care, the literature indicates that having a collaborative partnership with clients is clearly important to them and warrants a separate theme. Clients described power differentials in the care relationship, including providers exerting control over daily tasks, and dictating care, which contributed to low levels of autonomy and personal fulfilment (Doyle, 2012). The feeling of being ‘screened’ to determine if they were eligible to receive care was deemed an imbalance in the care relationship (Day et al., 2018), as was feeling like the passive participant, becoming more of the helper to advise staff of what care needed to be done, and having to negotiate the delivery of their own care needs when receiving care (Petriwskyj et al., 2014). Janlöv et al. (2006) explored older peoples' experiences in decision‐making, by looking both at their participation and influence. That study strongly reflected the older persons' views of having to be just satisfied with the care, feeling exposed and insecure in the care relationship, and portraying a sense of power imbalance between themselves and the staff providing care. Clients reported needing to balance their relationship with care workers in the hope of gaining influence over the care they received, proving the imbalance of power yet again in the care relationship. Positive experiences reported by clients that met their health and care needs included having a relationship with their case managers that was based on equality and confidentiality, made them feel in control, safe, secure and supported (Spoorenberg et al., 2015). Clients described being empowered to participate in their own care by staff and being able to actively contribute as an important factor in the care partnership (Gill et al., 2011). Clients reported inequalities in receiving information about care services, describing situations of having to ‘pull’ information out of providers and not receiving information if they did not know questions to ask (Tiilikainen et al., 2019).

#### 3.3.5 Quality domain 5: Clear communication

Clear communication was reflected strongly throughout the literature across several areas including accessing care, choosing care including having choice and control over care provided, relating with care providers and understanding the purpose and benefits of receiving care. Clients reported wanting access to information about other client's experiences of receiving care from service providers to enable a more informed choice when choosing a care provider (Council on the ageing Australia—COTA, 2018). Sefcik et al. (2016) asked clients what information they wanted to receive to help them make an informed decision about community services following discharge from hospital. That study reported a preference to receive practical information including costs associated with the service provided and information about how the service related to their needs, as being the most important factor to clients. The provision of quality service was described by clients as one in which care was provided by staff with professional attitudes and good communication skills (Firbank, 2012). Clients described negative impacts of care including providers changing care plans without consultation, scheduling times to deliver care that were not communicated to them and changing care staff with whom clients had built significant relationships over time, without notifying the client themselves (Doyle, 2012). Several studies reported client's dissatisfaction with receiving large volumes of paperwork and information that was difficult to understand or not being provided with any information and having to find information themselves (Day et al., 2018; Fraser et al., 2014; Gill et al., 2017; Royal Commission Commonwealth of Australia, 2020; Tiilikainen et al., 2019).

## 4 DISCUSSION

This scoping review focused on clients as users of community aged care services, with the aim of gathering information on service quality attributes that clients viewed as important. A second aim was to further explore these quality attributes to identify quality domains that could potentially inform the development of quality indicators to measure the quality of a community aged care service, that truly reflects a client‐centred model. To the author's knowledge, this is the first scoping review that focuses on what clients report as important qualities of aged care services delivered in the community. In this scoping review, it was considered an essential criterion for the inclusion of a study to have an outcome measure reported that reflected the clients' perspective (i.e., satisfaction or participation or viewpoint or preference).

Thus, a unique aspect of this study was specifying evidence of the involvement of a client in a study as essential inclusion criteria. Additionally, the inclusion of ‘involvement of a client’ captured studies that described client's views, experiences and perspectives relating to a variety of important stages of a clients community aged care service experience including navigating the aged care system, understanding what care was available to them, getting through the assessment process that determined their eligibility for a service, negotiating how care was delivered to meet their specific needs, communicating with care staff and service providers and balancing both formal and informal care.

A study by Jones et al. explored important dimensions of home care quality in 34 local authorities in England. The results of this study showed a clients' satisfaction with the services they received, and services delivered at suitable times were two performance indicators that reflected quality. In addition, this study showed the underlying constructs of home care quality were satisfaction and suitable times. This study also looked at the variance within the satisfaction ratings of dimensions of home care quality and suggested overall service quality had four aspects including service quality, positive carer quality, negative carer quality and outcome (Jones et al., 2007). The scoping review conducted to inform the results of this paper has explored this topic further, such that, the results have identified five specific domains of quality that truly represent the clients' perspectives of quality community aged care services.

A scoping review was conducted by Santana and colleagues in 2018 which aimed to identify quality indicators that could be used to measure patient‐centred care across healthcare settings. This study found inconsistency in the literature on how quality indicators were presented and measured, resulting from a lack of definitions describing the quality indicators themselves. In contrast, this scoping review examined the literature by focussing on the client's viewpoint in the first instance, the outcome of which resulted in the establishment of clear definitions of the key quality measures (described as outcomes in the individual studies reviewed; Table 2), which then informed the definition of each quality domain from the client's perspective (Table 8). Furthermore, the five quality domains identified in the literature are reflected in all stages of a clients' service experience, thus, confirming the validity of the quality domains identified in this study (Table 8).

Person‐centred care was identified as a single‐quality domain, but it is also a dominant theme in quality healthcare. Person centredness is a well‐recognised term that is used globally in health and social care services with a focus on placing the person at the centre of the delivery of care.

Internationally, England and the United States have led the way in adopting person‐centred care, following significant policy reform. Both countries have mandated requirements for service providers to collect and publish data on patient experiences, which is attached to financial incentives for providers who achieve high measures (Australian Commission on Safety and Quality in Health Care, 2011). It is not only recognised as important by organisations who deliver care but also by clients who receive care services. It is acknowledged that person‐centred care has a significant impact on a persons' experience and has been linked to improved outcomes (Miller & Peck, 2019). Zhou et al. (2021) and his colleagues found perceived provider compassionate care (described as the provider being interested in the persons' feelings, perspectives and experiences) and perceived provider shared decision‐making (described as the provider involving the person in treatment options available) resulted in increased satisfaction that directly correlated with a providers' communication. While that study focussed on young adults with upper respiratory tract infections seeking antibiotic medication in a primary care setting, it strongly suggests that person‐centred communication has an important impact on outcomes. Those results clearly identified that satisfaction was not merely related to the prescription of the antibiotic itself, but more so to effective communication displaying compassionate care provided by the treating physician, and the shared decision‐making approach taken. Person‐centred care adopted in primary healthcare has also demonstrated significant benefits including, a persons' ability to better manage their health (when they are informed and supported to do so), a reduced need to access speciality care, reduced hospitalisation rates and an increase in a persons' self‐perception and empowerment (Delaney, 2018). Given the dearth of papers from specific countries internationally (except Australia, *n* = 22), it is difficult to say more about specific countries and their aged care systems (All countries have eight or less papers).

In Australia, consumer directed care is a legislated requirement in the delivery of government‐funded community aged care services. It was introduced as an approach that enabled the consumer (client), to feel empowered and have influence over the care and services they receive (Mc Callum & Rees, 2017). A systematic review of consumer directed care for older people was undertaken by Ottmann et al. (2013), which aimed to identify user preferences for, and satisfaction with, community aged care services, to inform policymakers regarding future directions of consumer directed care models. Their results showed older people wanted greater involvement in care‐related decision‐making as well as greater choice and flexibility over how their care was delivered. The study also identified the differences between the preferences of older people in comparison to younger people with disabilities and those with mental health issues, highlighting the importance of future research to focus on what matters most to older people. Involving older people more directly in research is one way this might be achieved.

It is recognised that older Australians prefer to remain living in their homes rather than moving into residential aged care (Australian Government Productivity Commission, 2021). Measuring the quality of community aged care services is even more important now, not only from the end user's perspective but to also inform stakeholders (including organisations and governments) about the affordability, sustainability and efficacy of such services. In Australia, there is a gap in client‐driven quality measures that measure the appropriateness of services in addressing a client's need. Given this is one of the three main objectives set out by the Australian Government to measure aged care services (Australian Government Productivity Commission, 2021), it is now even more important that the opinions and preferences of clients about the aged care services they receive, be included when developing such a measure. The quality domains identified in this scoping review provide valuable information that could be used to inform future quality measures to meet government objectives.

## 5 STRENGTHS AND LIMITATIONS

One possible limitation of this review was the exclusion of articles published in languages other than English, which may have resulted in some important evidence being missed and which may have potentially influenced our conclusions. Nevertheless, this review was completed using a systematic approach including the clear identification of all relevant search terms and a thorough and comprehensive search of key databases to ensure all relevant articles were identified and included. In addition, the abstracts of relevant articles were reviewed by one reviewer and the full text was reviewed by two reviewers independently to confirm agreement regarding inclusion/exclusion. Although the data analysis was undertaken by one author only and is another potential limitation, its impact was mitigated by the other authors checking the results. Overall, we are confident that the review provides a comprehensive summary of the opinions and views of older people regarding quality community service, and that the identified quality domains are based on sound evidence.

Finally, a small number of articles (*n* = 3) that reported the preferences and experiences of older people living with cognitive impairment were identified and included in the review. Under‐representation of this client group makes it difficult to draw conclusions from the results that accurately reflect their perceptions regarding a quality aged care service. If their views are to be included in the development of these quality measures, further research to better understand their views is required. In addition, the number of articles that focussed on the perspectives and experiences of Aboriginal and Torres Strait Islander people when receiving community aged care services was limited (*n* = 5). Similarly, the number of articles that involved clients in the development of questionnaires and surveys used to understand a client's experience, satisfaction and preference were also limited. Thus, their opinions may not have been completely captured, resulting in a narrow representation of experiences and preferences that clients view as quality attributes of community aged care services. This shortcoming is currently being addressed through an increasing emphasis on the importance of including consumers in research activity that impacts them.

## 6 CONCLUSION

The five quality domains identified in this review that reflect clients' views of important attributes of a quality community aged care service provide an opportunity for community aged care organisations to explore how these could be incorporated to provide clients with a high‐quality service. These quality domains could be used to inform the development of quality indicators to measure the quality of aged care services from a clients' perspective.

## AUTHORS CONTRIBUTION

Sandra Smith was responsible for undertaking the literature searching and preparing the initial draft of the manuscript. All authors assessed the articles against inclusion and exclusion criteria, proofread and revised the manuscript.

## CONFLICT OF INTERESTS

None.

## Data Availability

The authors will make the data available upon reasonable request.
